# What Is Truth?

**Published:** 1917-03-31

**Authors:** 


### WHAT IS TRUTH?

It has been the invariable practice of The Hospital to correct any error which may have inadvertently appeared in its columns. For many years, as opportunity offered, we have co-operated with the Editor of *Truth*, in exposing abuses and grave defects in institutional methods, with gratifying results from a public point of view. We regret the article in our issue of March 17, page 469, contained the statement that "our contemporary, *Truth*, has entirely reversed its policy since the death of its founder, Mr. Henry Labouchere." The writer of the article, like ourselves, was under the impression that this was the case, but the policy in question was commenced during the lifetime of Mr. Labouchere, with his consent and knowledge. A portrait of Mr. Labouchere, supplied by him for the purpose, appeared repeatedly in advertisements based upon a Supplement which appeared in *Truth* in 1909. We therefore spontaneously withdraw the statement in question. That statement is not the fact, and we desire to make every possible amends to our contemporary for having inadvertently given publicity to it.

Our readers have known from time to time of the various cases in which The Hospital has co-operated for the public good in securing exposure for matters and persons where such action has been called for in the interests of the public. In this connection it may interest them to have before them the following facts. The present Editor of *Truth*, in an article, "Forty Years in *Truth's* Office," writes: For nearly three and a half years Mr. Labouchere, working as his own master, wrote every week columns of paragraphs and articles on every conceivable subject, doing, in addition, his own dramatic criticism and sometimes the City article. "The outflow from his pen was always in the highest degree entertaining, piquant, and original, and could not fail to make the fortune of any journal as long as Labouchere stuck to it." In 1880 Labouchere was returned for Northampton, and he changed his love from journalism to politics. He had been fortunate in finding Horace Voules to look after the business side of *Truth*, and "Voules used to say that from the very first he found that he had to be quite as much an assistant editor as a business manager. In particular, it was absolutely necessary to read all the proofs, because the Editor would not read any but his own, unless he detected something in them that appealed to him."

The present Editor, Mr. R. A. Bennett, began writing for *Truth* in 1884, and at Voules' suggestion entered *Truth's* office in 1889, and worked with Voules until his death. After it occurred Labouchere associated Mr. Bennett with himself in the management of the paper, and eight years ago Mr. Bennett succeeded to the editorship, and has been the responsible Editor of *Truth* ever since. Labouchere was a keen critic, and he once gave this as his instruction in the art of editing a newspaper: "You should find two or three fellows, who can turn out the right sort of stuff, and then leave them alone. I always found that if I had got to explain to a man the sort of article I wanted, and then go over his proof to knock it into shape, it was less trouble to write the thing myself." This was Labouchere's policy, and nothing could be written which could more forcibly demonstrate the ability of those responsible for the management and editing of *Truth* after Labouchere's personal retirement from these duties, than the success which attended the application of his policy to his own paper, for it yielded him some £300,000 from the time he established it until his death. Is there a newspaper proprietor who does not wish that he may be fortunate enough to enjoy an equal yield and success from his property, to that Mr. Labouchere experienced in the case of *Truth*?

